# Transcriptomic annotation of the Chungtien schizothoracin (*Ptychobarbus chungtienensis*) using Iso-seq and RNA-seq data

**DOI:** 10.1038/s41597-024-03491-x

**Published:** 2024-06-14

**Authors:** Zhendong Gao, Yuqing Chong, Ying Lu, Shiguang Ma, Zhen Wang, Jieyun Hong, Jiao Wu, Mengfei Li, Dongmei Xi, Weidong Deng

**Affiliations:** 1 Yunnan Provincial Key Laboratory of Animal Science and Feed, Faculty of Animal Science and Technology, Yunnan Agricultural University, Kunming, 650201 People’s Republic of China; 2 Diqing Tibetan Autonomous Prefecture Institute of Animal Husbandry and Veterinary Science, Shangri-La, 674499 People’s Republic of China

**Keywords:** Animal breeding, Gene expression

## Abstract

The Chungtien schizothoracin (*Ptychobarbus chungtienensis*), an endangered fish species endemic to the Zhongdian Plateau, remains underexplored in terms of transcriptomic sequencing. This investigation used tissues from five distinct organs (heart, liver, spleen, kidney, and brain) of the Chungtien schizothoracin for PacBio Iso-seq and RNA-seq analyses, yielding a repertoire of 16,598 full-length transcripts spanning lengths from 363 bp to 7,157 bp. Gene family clustering and phylogenetic analysis encompassed a comprehensive set of 13 fish species, all of which were cyprinids, including the zebrafish and the examined species Ptychobarbus chungtienensis. Moreover, the identification of long non-coding RNAs (lncRNAs) and coding sequences was accomplished across all five tissues. Comprehensive analyses of gene expression profiles and differentially expressed genes among the above five tissues were performed. In summary, the obtained full-length transcripts and detailed gene expression profiles of the Chungtien schizothoracin tissues furnish crucial expression data and genetic sequences, laying the groundwork for future investigations and fostering a holistic comprehension of the adaptive mechanisms inherent in the Chungtien schizothoracin under various conditions.

## Background & Summary

The Chungtien schizothoracin (*Ptychobarbus chungtienensis*) is exclusively found inhabiting the Zhongdian Plateau, which sits at an elevation of approximately 3,568 meters above sea level within the Tibetan Plateau, along with its adjacent aquatic habitats^1–3^. Owing to its delimited habitat range and heightened susceptibility to environmental shifts compared to fish species in low-altitude areas, the Chungtien schizothoracin species has developed adaptive mechanisms to overcome extreme conditions like low oxygen and low temperatures in high-altitude environments^4–7^. These distinctive traits make them an invaluable cohort for delving into the intricacies of fish acclimatization to elevated altitudes. Remarkably, the China Species Red List designates the Chungtien schizothoracin as an endangered species, highlighting its significance on a global scale^2,8^. In contrast to the extensive genomic and transcriptomic investigations of other fish species^9–12^, research on the Chungtien schizothoracin remains relatively limited, resulting in a scarcity of information concerning its full-length gene sequences.

The advent of advanced sequencing technologies, particularly global transcriptome analysis, has substantially propelled genetic investigations in fish, providing a fundamental underpinning for unraveling gene function and structure in non-model organisms without reference genome information^13–15^. However, a comprehensive exploration of transcriptomic and genomic landscapes in the Chungtien schizothoracin has not been fully explored, contributing to a deficiency in genetic insights crucial for understanding the adaptive mechanisms of this cherished fish. this fish species implies that there are certain physiological or behavioral adaptations in the Chungtien schizothoracin that are not fully understood at the molecular level. By studying the genetic information and gene expression patterns of this species, researchers aim to uncover the molecular basis of these adaptations, such as responses to high altitude environments, temperature fluctuations, oxygen levels, or food availability. Therefore, transcriptome sequencing emerges as a potent tool poised to swiftly enrich our comprehension of the functional genomics inherent to the Chungtien schizothoracin.

In this investigation, a diverse array of tissues, encompassing the heart, liver, spleen, kidney, and brain, extracted from the Chungtien schizothoracin, served as the substrate for integrated PacBio Iso-seq and RNA-seq analyses. The outcome yielded a substantial collection of 16,598 full-length transcripts, exhibiting a considerable length spectrum from 363 bp to 7,157 bp. Through meticulous bioinformatics scrutiny, it was discerned that 15,783 and 14,064 transcript sequences found alignment within the NCBI non-redundant protein sequences (Nr) and Swissprot database, respectively. Further annotation unveiled 14,690, 13,609, and 11,406 transcripts affiliating with the Kyoto Encyclopedia of Genes and Genomes (KEGG), euKaryotic Ortholog Groups (KOG), and Gene Ontology (GO) databases, respectively. Extending beyond mere coding sequences, the exploration also encompassed the identification of long-chain non-coding RNAs (lncRNAs) across all the sampled tissues from the Chungtien schizothoracin. The culmination of these findings, encapsulated in the full-length transcripts and gene expression profiles, posits itself as a trove of genetic information. This transcription repository not only paves the way for comprehensive functional genomic inquiries but also lays the foundation for an enriched understanding of the adaptive mechanisms governing the survival of the Chungtien schizothoracin.

## Methods

### Ethics statement

All fish handling and experimental procedures in this study were approved by the Animal Care and Use Committee of Yunnan Agricultural University (Approval Code: 202212001, Approval Data: 1 December 2022).

### Collection of samples and preparation of RNA

A female sampling individual was officially fished from the Aquatic Biological Resources Survey conducted by the Diqing Prefecture Institute of Animal Husbandry and Veterinary Science (submitted from the Luoji Section of Luoji River in Shangri-La City). It was 4–6 months old and a quintet of tissues (heart, liver, spleen, kidney, and brain) underwent prompt cryopreservation in liquid nitrogen. The extracted RNA sample is then added to DNaseI buffer, followed by the addition of an appropriate amount of DNaseI enzyme. The RNA sample is subjected to DNaseI digestion at 37 °C for 30 minutes. Subsequently, DNaseI stop solution is added to ensure complete removal of DNaseI enzyme. Finally, RNA purification and detection are carried out to ensure that the RNA sample does not contain any residual DNA. TRIzol reagent (Invitrogen) facilitated individual RNA isolation from each tissue following the manufacturer’s protocol. Initial assessment of RNA concentration and purity employed Nanodrop 2000, while agarose gel electrophoresis scrutinized genomic contamination, purity, and RNA integrity. Subsequently, the RIN (RNA Integrity Number) value was meticulously determined using the Agilent 2100 platform.

### PacBio library construction and sequencing

For the construction of the PacBio sequencing library, equal proportions of qualified RNA from the five tissues was pooled together for library construction at Wuhan Frasergen Gene Biotechnology Co., Ltd (Wuhan, China). Eukaryotic mRNA 3 ‘terminal has a poly-A tail structure, Primers with Oligo dT were used to pair A-T bases with poly-A as primers for reverse synthesis of cDNA, and primers were added to the end of full-length cDNA of reverse synthesis. The full-length cDNA was amplified by PCR, and the product was purified by PB magnetic beads to remove some small fragments of cDNA less than 1 kb. Repair the end and connect the SMRT dumbbell connector. The unconnected fragments were digested by exonuclease and purified by PB magnetic beads to obtain the sequencing library. After the library construction was completed, Qubit 3.0 was used for accurate quantification, and Agilent 2100 was used to detect the library size. Machine sequencing could be carried out only after the library size met expectations. The raw data retrieved from the sequencer underwent filtration and processing using SMRTlink 10.0 software, with the parameter–minLength = 50 configured to exclude sequences below 50 base pairs. Post this filtering process, the obtained data were considered valid.

### mRNA Library construction and sequencing

mRNA from the five tissues of the Chungtien schizothoracin was accomplished using Oligo(dT) magnetic beads. Total RNA was extracted using the Trizol (Invitrogen, CA, USA), RNA purity and integrity was monitored by NanoDrop 2000 spectrophotometer (NanoDrop Technologies, Wilmington, DE, USA) and a Bioanalyzer 2100 system (Agilent Technologies, CA, USA). RNA contamination was assessed by 1.5% agarose gel. Adhering to the manufacturer’s instructions, mRNA purification was executed, and libraries were constructed utilizing the V AHTS Universal V6 RNA-seq Library Kit for MGI (Vazyme, Nanjing, China). The Qubit 2.0 Fluorometer (Life Technologies, Carlsbad, CA, USA) and the Agilent 2100 system (Agilent Technologies, CA, USA) were employed to evaluate the quality and size of the libraries. Subsequently, sequencing was performed on the MGI-SEQ 2000 platform at Wuhan Frasergen Gene Biotechnology Co., Ltd (Wuhan, China).

### PacBio ISO-seq data processing

**Fig. 1:**
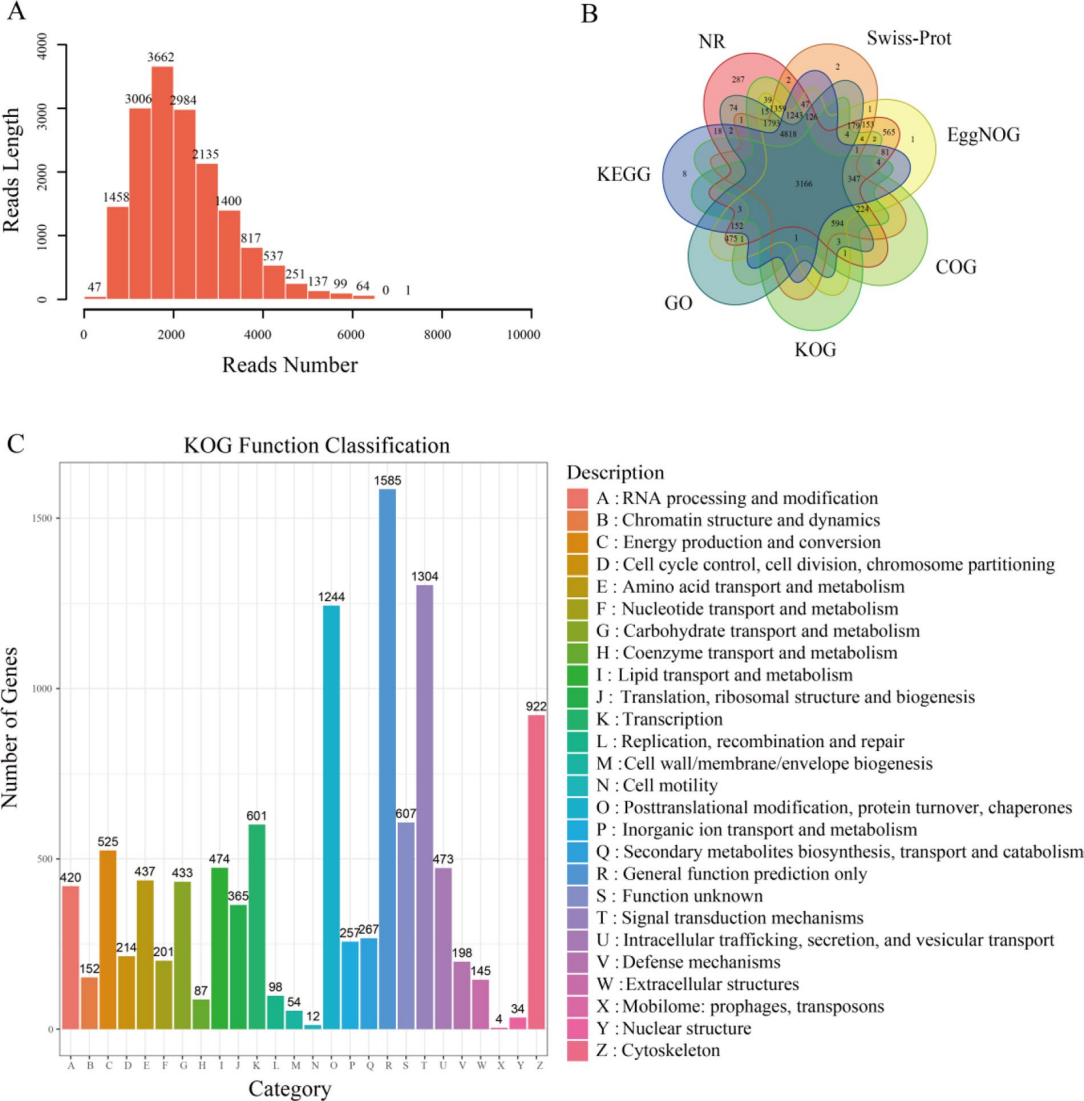
ISO-seq isoforms analysis and annotation in the Chungtien schizothoracin. (**A**). Length distribution of ISO-seq isoforms. (**B**). Venn diagram analysis of ISO-seq isoforms against NR, KOG, COG, Swiss-Prot, EggNOG, GO and KEGG. (**C**). KOG function analysis of ISO-seq isoforms.

As delineated in Table 1, PacBio ISO-seq yielded a comprehensive 20,724,543 subreads. The ccs tool was deployed to derive Circular Consensus Sequences (CCS) from subreads, employing the–all parameter to retain all quality CCS, culminating in 396,323 CCS. Subsequent refinement, conducted through the limma tool, entailed the removal of chimeric and polyA-tailed sequences, resulting in a robust set of 261,878 Full-Length Non-Chimeric (FLNC) reads, boasting an average length of 2,133 bp. IsoSeq 3 played a pivotal role in clustering and deduplicating reads, forming consensus sequences by aggregating akin sequences and optimizing transcripts. These optimized transcripts underwent correction and deduplication through LoRDEC, ultimately yielding a collection of 16,598 high-quality consensus isoforms, exhibiting an accuracy rate surpassing 99% and an average length of 2,207.3 bp (Fig. 1A). The non-redundant full-length transcript dataset exhibited exceptional integrity, serving as the foundation for subsequent analyses.

**Table 1:** Statistic of ISO-sequencing in the Chungtien schizothoracin.

Type	Total bases (bp)	Total reads number	Average length (bp)
Subreads	39.4 G	20,724,543	1,904
CCS	992,873,706	396,323	2,505
FLNC	558,757,016	261,878	2,133
Refined	503,226,762	247,582	2,032
Before_correct	36,721,664	16,598	2,212
After_correct	36,636,766	16,598	2,207

### Function annotation of unigenes

Functional Annotation of Unigenes in the Chungtien schizothoracin ensued through a meticulous comparison of identified Unigenes against seven databases, namely NR, NT, Swiss-Prot, Pfam, GO, KEGG, and COG. For the NT database analysis, the BLAST software with an E-value < 1 × 10^−10 was deployed. As for NR, COG, and Swiss-Prot annotations, the Diamond BLASTX^16^ method, with an E-value < 1 × 10^−10, was harnessed for in-depth analysis. The Pfam database underwent scrutiny through the Hmmscan program, and the foundation for GO annotation analysis was derived from NR alignment results. The KEGG functional categories underwent comprehensive analysis through the KofamKOALA method (https://www.genome.jp/tools/kofamkoala/).

### Quality control of annotation

**Fig. 2:**
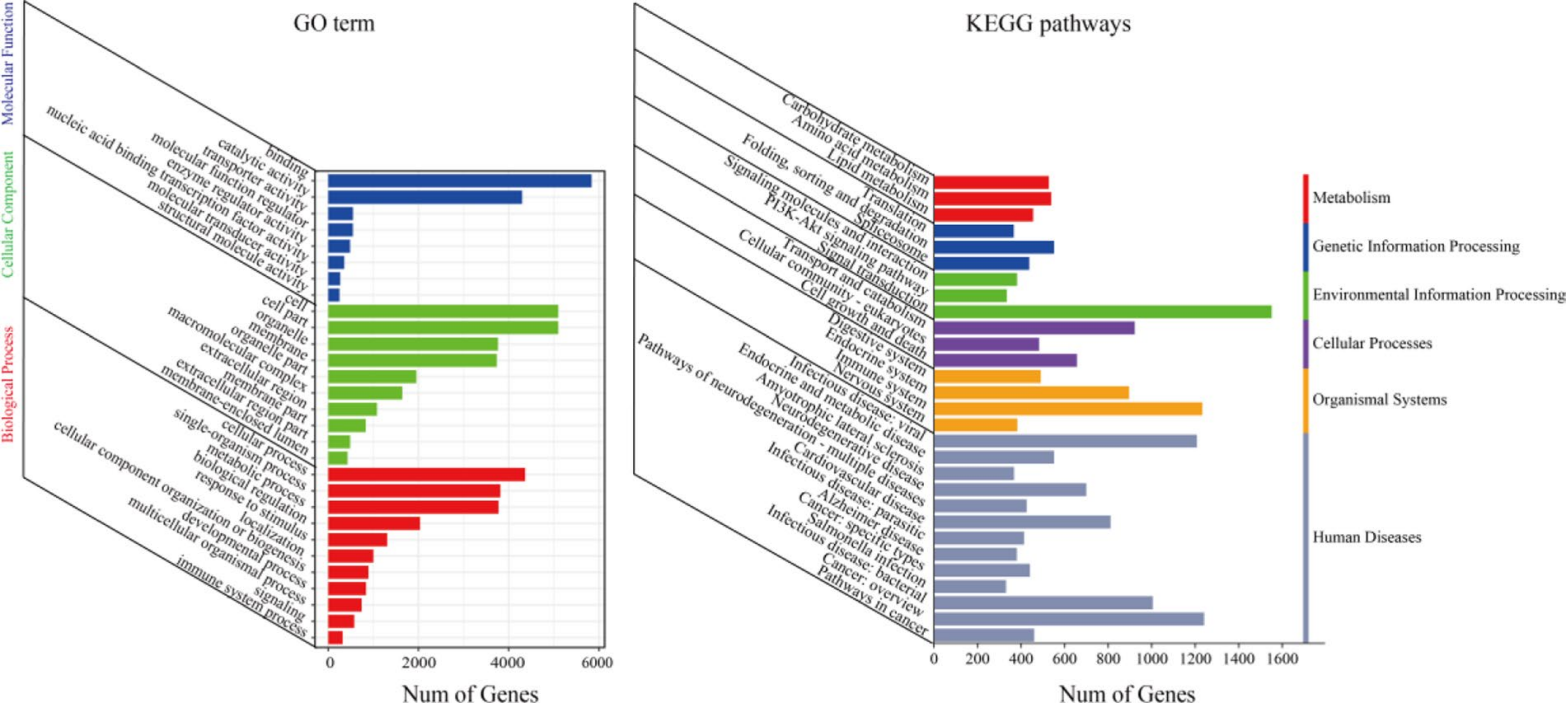
GO ontology (**A**) and KEGG pathways (**B**) analysis for the annotated sequences of the Chungtien schizothoracin.

Quality Control of Annotations was undertaken through a comprehensive examination of full-length transcripts against multiple reference databases. Initially, within the NCBI non-redundant protein sequence (Nr), KOG, Swiss-Prot, EggNOG, and COG databases, 15,783 (95.09%), 13,609 (81.99%), 14,064 (84.73%), 15,401 (92.79%), and 4,347 (26.19%) transcripts, respectively, exhibited concordant sequences. Additionally, 11,406 (68.72%) and 9,462 (60.34%) transcripts found annotation in the KEGG and GO databases, respectively (Fig. 1B). Furthermore, ISO isoforms underwent mapping to the KOG database for rigorous functional classification (Fig. 1C). The analysis of GO ontology and KEGG pathways (Fig. 2) was conducted using TBtools-II^17^, facilitating the identification of annotated sequences and the exploration of active biological pathways in the Chungtien schizothoracin. For instance, KEGG analysis unveiled that Unigenes predominantly partake in six pathways, metabolism, genetic information processing, environmental information processing, cellular processes, and organismal systems, human diseases. Furthermore, we utilized the TransDecoder software to prognosticate and scrutinize the coding sequences of the Chungtien schizothoracin.

**Fig. 3:**
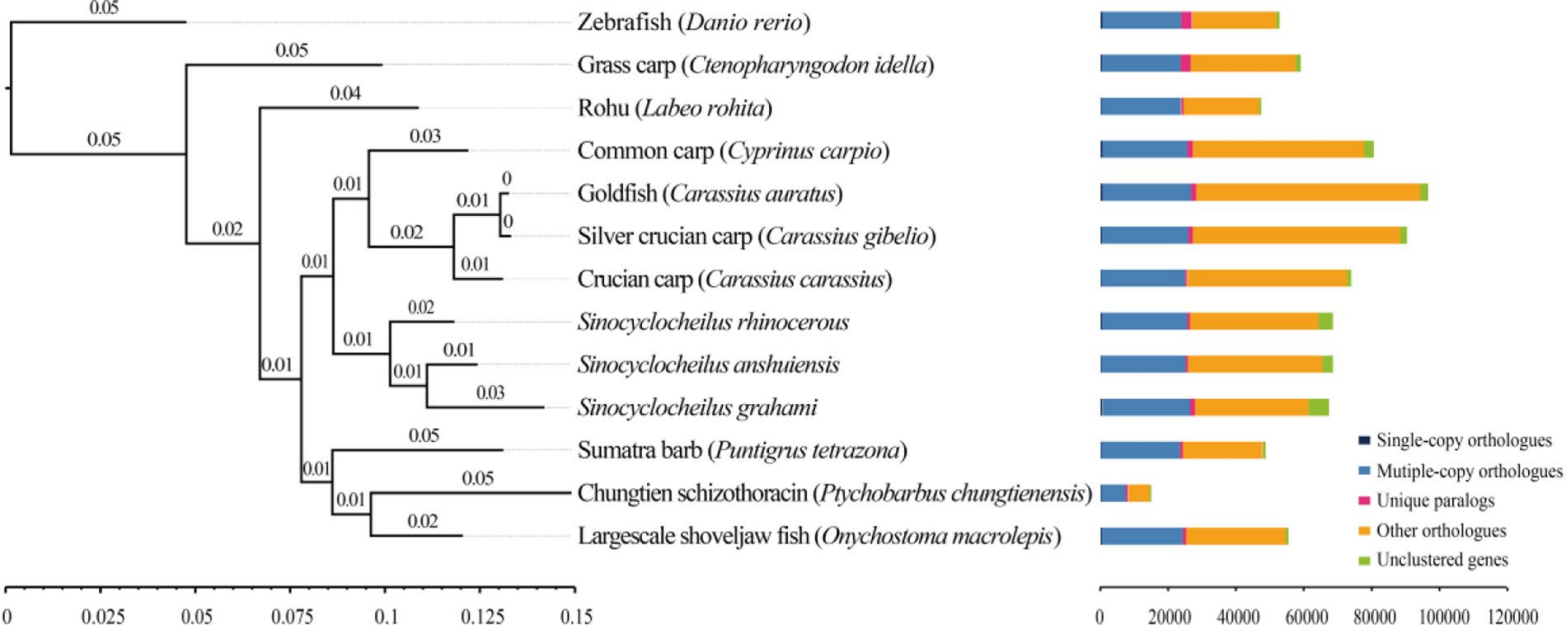
Cluster analysis of gene families and phylogenetic analysis. Cluster analysis of gene families of 13 fish species and phylogenetic analysis of 13 fish species though a rooted STAG species phylogenetic tree using the maximum likelihood method.

For clustering analysis based on sequence similarity (Fig. 3A), the protein sequences of 14 species, as later enumerated, were instrumental. All alignments adhered to an E-value threshold of <1e−5. As depicted in Fig. 3A, a total of 14,910 genes were unveiled in the Chungtien schizothoracin, showcasing multiple homologous genes clustered among the 13 fish species, comprising 12 cyprinid species and the zebrafish. Subsequent to this, a phylogenetic tree was meticulously constructed deploying OrthoFinder with the maximum likelihood method (Fig. 3B). The outcomes elucidated that the Chungtien schizothoracin shares close affinities with Onychostoma macrolepis, a multi-scaled scraper in the Cyprinidae family. In a holistic context, the phylogenetic relationships impeccably align with the classification and evolutionary standing of these species.

**Fig. 4:**
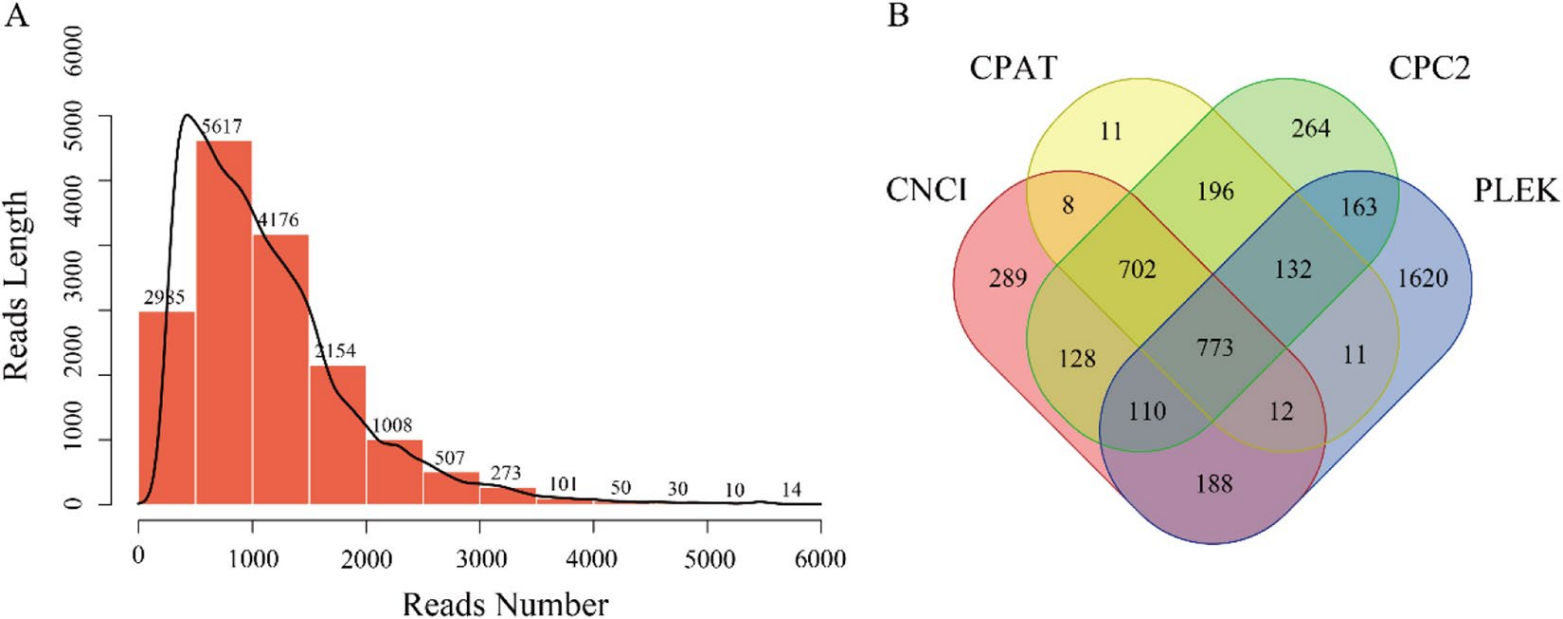
Function prediction of ISO-seq isoforms in the Chungtien schizothoracin. (**A**). CDS prediction of ISO-seq isoforms. (**B**). Venn diagram analysis of predicted lncRNAs of ISO-seq isoforms.

Additionally, we predicted the coding sequence (CDS) of all full-length transcripts. As illustrated in Fig. 4A, the average lengths of CDS in the Chungtien schizothoracin are primarily distributed around 1,152 bp. The longest and shortest lengths of CDS are 5,805 bp and 279 bp, respectively. Long non-coding RNAs (lncRNAs) play a crucial role in the regulation of growth and development in many fish species. Here, potential lncRNAs were predicted using CPC2, CPAT, PLEK, and CNCI databases. As depicted in Fig. 4B, the Venn diagram analysis of predicted lncRNAs from these four software packages revealed a total of 773 identified lncRNAs.

### Predictions of open reading frames (ORFs) and long non-coding RNAs (lncRNAs)

The prediction of open reading frames (ORFs) within the Chungtien schizothoracin transcripts was conducted using the TransDecoder v2.0.1 package (https://transdecoder.github.io/). Transcripts possessing complete ORFs, along with 5′ and 3′ untranslated regions, were identified as full-length transcripts. To predict long non-coding RNAs (lncRNAs), the CNCI^18^, CPC2^19^, CPAT^20^, and PLEK^21^ software tools were employed.

### Identification of differentially expressed genes in the different tissues

Correlation analysis and calculation of RPKM (Reads Per kb per Million reads) values were employed to ascertain the gene expression levels across the 5 tissues of the Chungtien schizothoracin. Differentially expressed genes (DEGs) were identified based on a log-fold expression change (log FC) greater than 2 or less than −2, utilizing a threshold for false discovery rates (FDR < 0.001) and a statistically significant P value (P < 0.005).

### Cluster analysis of gene families and phylogenetic analysis

Performing cluster analysis based on sequence similarity, protein sequences from 13 fish species, comprising 12 cyprinid species and the zebrafish (*C. auratus*, *C. carassius*, *C. carpio*, *C. gibelio*, *C. idella*, *D. rerio*, *L. rohita*, *O. macrolepis*, *P. chungtienensis*, *P. tetrazona*, *S. anshuiensis*, *S. grahami*, *S. rhinocerous*) were utilized. The analysis encompassed the removal of redundancy and alternative splicing, retaining only the longest transcripts. Multiple sequence alignment was carried out with the “-S diamond” option, and ultimately, the analysis incorporated the “-M msa -T iqtree” parameters to construct a rooted STAG (Species Tree Inference from All Genes) species phylogenetic tree using the maximum likelihood method^22^.

## Data Records

All RNA-seq raw reads of the Chungtien schizothoracin were deposited in the Sequence Read Archive (SRA) of the National Center for Biotechnology Information under accession number SRR28201811, SRR28201812, SRR28201813, SRR28201814, SRR28201815, SRR28201816^23–28^. Also, all raw full-length Iso-seq reads of the Chungtien schizothoracin were deposited in the Sequence Read Archive (SRA) of the National Center for Biotechnology Information under accession number SRR28340047^29^.

## Technical Validation

**Fig. 5:**
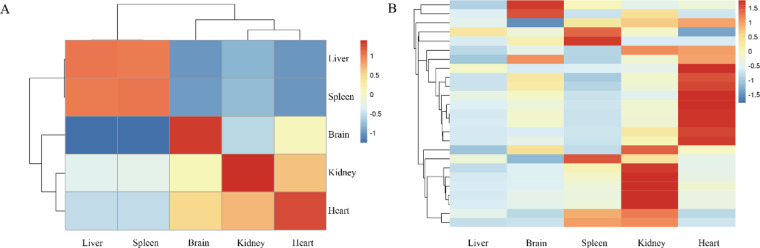
Gene expression profile in 5 tissues (heart, liver, spleen, kidney, and brain) in the Chungtien schizothoracin. (**B**) Heatmap analysis of the differentially expressed mRNAs in 5 tissues.

To elucidate the global mRNA expression patterns in the Chungtien schizothoracin, samples from five tissues (heart, liver, spleen, kidney, and brain) were subjected to sequencing. Evaluation of RNA-seq clean reads quality was conducted using FastQC, revealing an average mapping rate (clean reads/original reads) of 98.2%. The average Q20 and Q30 were 96.4% and 88.6%, respectively. The GC content distribution in the Chungtien schizothoracin tissue samples exhibited a normal pattern, indicating the absence of sequencing data contamination. Given the lack of a reference genome for the Chungtien schizothoracin, alignment of clean reads from the five tissue samples was performed against the isoforms generated by PacBio ISO-seq. Subsequent correlation analysis (Fig. 5A) and examination of gene expression profiles across the five tissues (Fig. 5B) provided insights into the mRNA expression dynamics of the Chungtien schizothoracin.

## Data Availability

All software utilized in this study is publicly available, and their parameters are explicitly outlined in the Methods section. In instances where specific parameters for the software were not detailed, default settings, as recommended by the developers, were employed.
